# H2B ubiquitylation is part of chromatin architecture that marks exon-intron structure in budding yeast

**DOI:** 10.1186/1471-2164-12-627

**Published:** 2011-12-22

**Authors:** Grace S Shieh, Chin-Hua Pan, Jia-Hong Wu, Yun-Ju Sun, Chia-Chang Wang, Wei-Chun Hsiao, Chia-Yeh Lin, Luh Tung, Tien-Hsien Chang, Alastair B Fleming, Cory Hillyer, Yi-Chen Lo, Shelley L Berger, Mary Ann Osley, Cheng-Fu Kao

**Affiliations:** 1 Institute of Statistical Sciences, Academia Sinica, Academia Road, Nankang, Taipei 115, Taiwan; 2 Institute of Cellular and Organismic Biology, Academia Sinica, Academia Road, Nankang, Taipei 115, Taiwan; 3 Genomic Research Center, Academia Sinica, Academia Road, Nankang, Taipei 115, Taiwan; 4 Institute of Food Science and Technology, National Taiwan University, Daan, Taipei 106, Taiwan; 5 Microbiology, Moyne Institute of Preventive Medicine, The University of Dublin, Trinity College, College Green, Dublin2, Dublin, Ireland; 6 Departments of Cell and Developmental Biology, University of Pennsylvania, Philadelphia, PA 19104, USA; 7 Molecular Genetics and Microbiology, University of New Mexico School of Medicine, Albuquerque, NM 87131, USA

## Abstract

**Background:**

The packaging of DNA into chromatin regulates transcription from initiation through 3' end processing. One aspect of transcription in which chromatin plays a poorly understood role is the co-transcriptional splicing of pre-mRNA.

**Results:**

Here we provide evidence that H2B monoubiquitylation (H2BK123ub1) marks introns in *Saccharomyces cerevisiae*. A genome-wide map of H2BK123ub1 in this organism reveals that this modification is enriched in coding regions and that its levels peak at the transcribed regions of two characteristic subgroups of genes. First, long genes are more likely to have higher levels of H2BK123ub1, correlating with the postulated role of this modification in preventing cryptic transcription initiation in ORFs. Second, genes that are highly transcribed also have high levels of H2BK123ub1, including the ribosomal protein genes, which comprise the majority of intron-containing genes in yeast. H2BK123ub1 is also a feature of introns in the yeast genome, and the disruption of this modification alters the intragenic distribution of H3 trimethylation on lysine 36 (H3K36me3), which functionally correlates with alternative RNA splicing in humans. In addition, the deletion of genes encoding the U2 snRNP subunits, Lea1 or Msl1, in combination with an *htb-K123R *mutation, leads to synthetic lethality.

**Conclusion:**

These data suggest that H2BK123ub1 facilitates cross talk between chromatin and pre-mRNA splicing by modulating the distribution of intronic and exonic histone modifications.

## Background

Genome-wide histone modification maps have now been generated for a number of eukaryotic organisms. These maps have revealed the preferential localization of specific marks to active or silent chromatin and the association of marks of active transcription with different regions of genes. For example, H3 trimethylation on lysine 4 (H3K4me3) is enriched at the 5' ends of actively transcribed genes while H3 trimethylation on lysine 36 (H3K36me3) is localized towards the 3' ends of coding regions. These localization patterns are related to the roles that the marks play in transcription: H3K4me3 regulates the efficiency of transcription initiation and early steps in transcription elongation, and H3K36me3 prevents the utilization of cryptic initiation sites in coding regions and controls aspects of transcription termination and processing [1-4]. Most eukaryotic genes are modular, containing multiple exons interrupted by introns. Genome-wide histone modification maps from *C. elegans *and human revealed that intron-exon chromatin is also preferentially marked, with transcriptionally active modifications generally excluded from introns and concentrated in exons [5-11]. These studies concluded that this pattern was primarily the consequence of different levels of nucleosome occupancy in these regions because nucleosomes were depleted in introns relative to exons. However, a recent analysis of published human epigenomic data found that 10 histone modifications were enriched in the 5' introns of human genes independently of the level of nucleosome occupancy [12]. It was suggested that the presence of these marks reflects an aspect of the splicing process such as exon definition and could play a direct role in regulating splicing. Thus, the location of intragenic histone modifications and the functional roles associated with different localization patterns remain areas of intense investigation.

One important intragenic histone modification is the monoubiquitylation of H2B (H2BK123ub1). H2B is ubiquitylated co-transcriptionally and in turn regulates the presence of other active chromatin marks during the transcription process, including H3K4, H3K36, and H3K79 methylation [13-19]. The presence of H2BK123ub1 in chromatin has been associated with both nucleosome stabilization and destabilization. H2BK123ub1 and the histone chaperone, Spt16, have been shown to function interdependently during transcription elongation to regulate nucleosome reassembly and preserve chromatin integrity [20-22]. Biochemical evidence and genomic nucleosome occupancy data also indicate that the presence of H2BK123ub1 generally promotes nucleosome stability [23,24]. However, it was recently shown that synthetic nucleosome arrays containing H2BK123ub1 are less compact and exhibit an increase in inter-nucleosomal distance, as compared to arrays containing unmodified H2B [25]. In addition, two recent reports described a putative role for this modification in mediating chromatin decondensation at DNA damage sites [26,27]. Thus, H2BK123ub1 may differentially affect chromatin structure in a context dependent manner.

In this report, we generated a genome-wide map of H2BK123ub1 occupancy in budding yeast to determine if the distribution of this modification could be related to additional biological processes. We found that H2BK123ub1 was enriched across gene coding regions and marked both introns and exons of ribosomal protein (*RP*) genes, and that the level of this mark was further increased at 3' intron-exon boundaries. The presence of H2BK123ub1 in introns of *RP *genes was separable from nucleosome occupancy, which was generally lower in introns compared to exons. In addition, we noted that disruption of H2B ubiquitylation tended to alter the distribution of H3K36 trimethylation in intragenic regions. H3K36me3 has been functionally linked to pre-mRNA splicing in worms and humans [6,7,28]. Furthermore, when an *htb-K123R *mutation was combined with deletions of *LEA1 *and *MSL1*, whose products facilitate U2 snRNA association with pre-mRNA [29,30], we found a synthetic lethal phenotype. These data suggest that by modulating the distribution of intronic and exonic histone modifications, H2BK123ub1 facilitates cross talk between chromatin and pre-mRNA splicing.

## Results

### H2B ubiquitylation is enriched in transcribed regions

**Figure 1:**
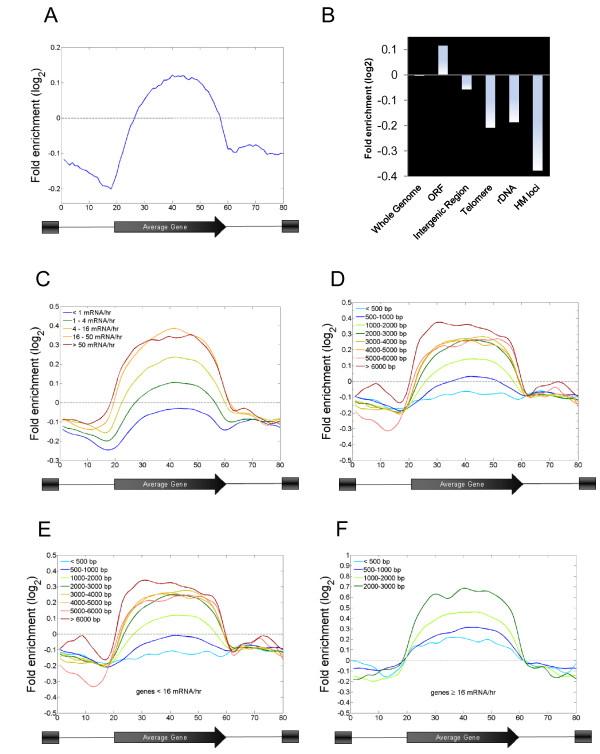
**H2B ubiquitylation is enriched at gene coding regions and correlates with transcriptional activity and gene length**. (A) H2BK123ub1 is enriched at gene coding regions. H2BK123ub1 ChDIP-chip was performed using a high-resolution oligonucleotide tiling array. The log2 ratio of ubiquitylated H2B in wild type yeast cells was subjected to the averaged gene analysis (detailed in Materials and Methods). (B) H2BK123ub1 levels at various genomic locations calculated as in (A). ORF: open read frame; intergenic region; telomere: the average of the last 20 Kb of each chromosome; rDNA: ribosomal DNA; *HM*: the silent *HMRa *and *HMLa *loci. (C) H2BK123ub1 levels are proportional to transcription rate. All yeast genes were divided into five subclasses according to transcription rate [35]. Composite H2BK123ub1 profiles for each subclass are shown on averaged genes based on the analysis from Pokholok *et al*. (2005). (D) H2BK123ub1 levels are correlated with gene length. All yeast genes were divided into 8 subclasses according to the length of their coding regions. Composite H2BK123ub1 profiles for each subclass are shown on averaged genes as in (C). (E) Genes with transcription rates below 16 mRNA/hour were selected and divided into subclasses based on gene length as in (D). The average enrichment of H2BK123ub1in each subclass was plotted as a function of the averaged genes. (F) Genes with transcription rates greater than or equal to 16 mRNA/hour were selected and divided into subclasses based on gene length as in (D) and then plotted as in (E).

Previous gene-specific studies in yeast showed that H2BK123ub1 is present at genomic regions that are actively transcribed and absent from transcriptionally silent chromatin [16,18,20,31-33]. To map the distribution of H2BK123ub1 across the yeast genome, we used the chromatin double immunoprecipitation or ChDIP technique [16,31] to detect HA-ubiquitin modified Flag-H2B combined with a high-resolution oligonucleotide-tiling array. We then calculated the level of H2BK123ub1 relative to the level of H2B to account for the effects of different histone density across the genome. H2BK123ub1 was found to be preferentially enriched across transcribed regions in yeast (Figure 1A), consistent with its postulated roles in transcriptional elongation [18,20,24,32]. These results are similar to those reported in a recent genome-wide study in humans using a monoclonal antibody against vertebrate H2BK120ub1 [34]. In contrast to its enrichment at coding regions, H2BK123ub1 was depleted at intergenic regions and in silent chromatin, including telomeric, rDNA and *HM *loci (Figure 1B; Additional file 1, Figure S1).

A recent ChIP-chip study using a polyclonal antibody directed against yeast H2BK123ub1 also found that this mark was enriched at gene coding regions [8]. We compared the two genome-wide data sets and found almost identical patterns of H2BK123ub1 localization at a number of different genomic locations, with somewhat higher levels of H2BK123ub1 detected by the ChDIP method (Additional file 1, Figures S2A, S3A, S4A) Thus, two different approaches to detect H2BK123ub1 lead to the conclusion that this mark is primarily associated with the transcribed regions of genes.

Next, we investigated the relationship between H2BK123ub1 levels and transcription rate. We found that the level of H2BK123ub1 at coding regions was proportional to transcription rate, with the highest levels occurring at genes that have a transcription rate of > 16 copies of mRNA per hour (Figure 1C; Additional file 1, Figure S1). Genes in this category represent 8.2% (389 genes) of the 4802 genes whose transcription is detectable in rich media (Additional file 1, Tables S1 and S2) [35]. The strong correlation between H2BK123ub1 levels in coding regions and transcriptional activity suggests that the majority of H2BK123ub1 in the yeast genome is established during transcription elongation through the association of the H2B ubiquitylation machinery with elongating RNA polymerase II [32].

Intriguingly, the level of H2BK123ub1 also showed a correlation with gene length, with higher levels of H2BK123ub1 present at genes with longer coding regions (Figure 1D). Long genes have a higher probability of containing cryptic transcription initiation sites in their coding regions, and increased levels of H3K36 methylation at these genes have been correlated with the suppressed utilization of these sites [1,36]. In the case of H3K36 methylation, increased levels of the modification are found predominantly at long genes that are infrequently transcribed [36]. However, H2BK123ub1 is present at long genes with both low and high rates of transcription (Figure 1E, F; Additional file 1, Table S2). We have previously shown that H2BK123ub1 plays a role in restoring chromatin structure displaced by elongating RNAPII [19]. Thus, at long genes, the increased levels of H2K123ub1 could be important for preventing the utilization of cryptic transcription initiation sites by ensuring nucleosome reassembly. Conversely, at shorter genes, where suppression of cryptic transcription is not a general issue, the enrichment of H2BK123ub1 at coding regions could serve additional functions, such as regulating the levels of other transcription-related histone modifications or other events in the transcription process [4-18,33].

### Comparison of the genome-wide patterns of H2B ubiquitylation and H3K4me3, H3K79me2/3, and H3K36me3

Histone H3 is modified by methylation at lysines 4 and 36 at its N terminus and at lysine 79 in the globular domain of H3. These three H3 methylations have been functionally linked to the levels of H2BK123ub1, with H3K4 and H3K79 di- and tri-methylation levels reduced, and H3K36 tri-methylation levels enhanced, in the absence of H2BK123ub1 [15-19,37]. To investigate the links between H2BK123ub1 and these H3 methylations at the genomic level, we compared the map of H2BK123ub1 occupancy to published genome-wide maps of H3K4me3, K36me3 and K79me2/me3 methylation (Additional file 1, Figure S2, S3). By superimposing the genomic profiles of H2BK123ub1 and H3K4me3 [38] over the average gene and at individual chromosomal loci, we found that the two histone marks occupy distinct regions at yeast genes: H3K4me3 peaks at promoters and 5' coding regions, whereas H2BK123ub1 levels are very low at promoters and peak over coding regions (Additional file 1, Figure S2A, B).

Both H3K36me3 and H2BK123ub1 have been connected to the suppression of internal transcription initiation in gene coding regions [20,39,40], and the results discussed above indicate that the two marks are enriched at the coding regions of long genes. The genome-wide and regional distribution patterns of H2BK123ub1 and H3K36me3 are also similar, with each mark absent from transcription start sites and concentrated along coding regions (Additional file 1, Figure S2C, D). However, H3K36me3 is more enriched towards the 3' end of coding regions, consistent with its additional roles in 3' end processing [38]. Finally, a comparison of our H2BK123ub1 profile with a recent analysis of H3K79me2/me3 genome-wide occupancy [8] confirmed the high degree of co-localization between H2BK123ub1 and H3K79me3 over gene coding regions (Additional file 1, Figure S2E, F; Figure S3B; Figure S4C) and the general anti-correlation between the distribution of H2BK123ub1 and H3K79me2, which is enriched at intergenic regions (Additional file 1, Figure S4B). However, unlike H2BK123ub1, neither H3K79 methylation state correlates closely with transcription rate [8,38].

### H2BK123ub1 is present in introns and exons of *ribosomal protein *genes

**Figure 2:**
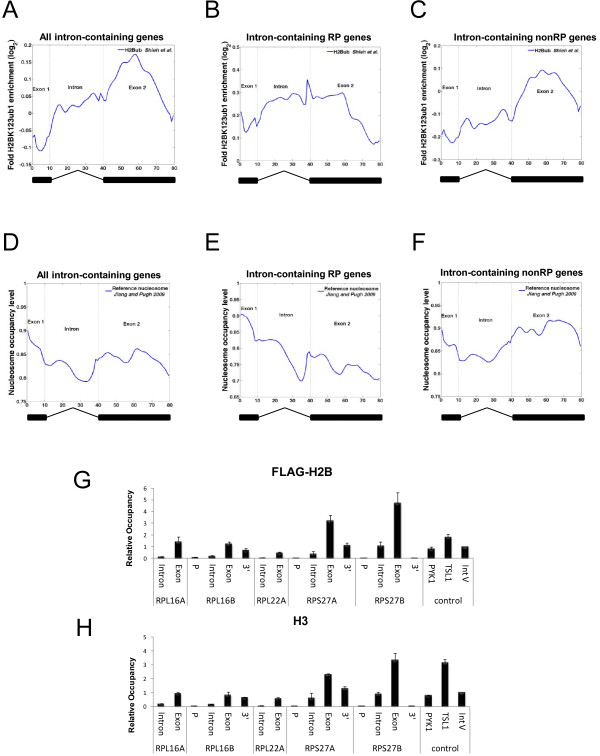
**H2B ubiquitylation marks introns and exons**. (A-C) Localization of H2BK123ub1 across the coding region of all intron-containing, *RP *and non-*RP *genes. The DNA sequences of exon1, intron and exon2 of all intron-containing genes in yeast were divided into 10, 30 and 40 bins, respectively. The composite of the averaged H2BK123ub1 level was aligned in the order: exon1-intron-exon2. (D-F) Nucleosome occupancy across the coding region of all intron-containing *RP *and non-*RP *genes. The composite profiles were analyzed as in (A-C). The average occupancy of nucleosomes in introns and exons was derived from data in Jiang and Pugh (2009) [42] and calculated as described in Materials and Methods. (G) H2B and (H) H3 occupancies at several intron- containing ribosomal protein (*RP*) genes were assayed by ChIP using an anti-Flag antibody to detect FLAG-H2B and an antibody against the C-terminus of H3. The relative H2B and H3 occupancies were calculated by comparison to the occupancy of these histones at the *INT-V *region.

Next, we sought to determine if the enrichment of H2BK123ub1 in coding regions was related to intron-exon architecture in budding yeast. In *Saccharomyces cerevisiae*, 280 genes are known to contain introns, and the majority of these genes contain a single intron that is flanked by a small 5' exon (as small as 1 bp) and a large 3' exon. We generated a fine structure map of H2BK123ub1 occupancy across the coding regions of these intron-containing genes, with the map of the average intron-containing gene shown in Figure 2A. The analysis showed that immediately after the 5' exon-intron boundary, which had a low level of H2BK123ub1, the level of this mark rose gradually and peaked toward the center of the 3' exon. In addition, the level of H2BK123ub1 in introns was lower than its level in exons. During the exponential growth of yeast cells, the highly transcribed ribosomal protein (*RP*) genes represent ~90% of pre-RNA splicing events [41]. We postulated that the average distribution of H2BK123ub1 in all intron-containing genes (Figure 2A) might not reflect the pattern of genes with high splicing activity. Thus, we separated the intron-containing genes into *RP *and non-*RP *genes and repeated the analysis of H2BK123ub1 distribution in these two classes of genes (Figure 2B, C). The analysis indicated that in both groups of genes the level of this mark rose immediately after the 5' exon-intron boundary and peaked close to the 3'intron-exon boundary (Figure 2B, C). However, the overall level of H2BK123ub1 in *RP *genes (Figure 2B) was significantly higher than in non-*RP *genes (Figure 2C). Importantly, similar levels of H2BK123ub1 were present in the introns and exons of *RP *genes (Figure 2B), while the level of H2BK123ub1 in the introns of non-*RP *genes was lower than in exons (Figure 2C), similar to the pattern of all intron-containing genes (Figure 2A). These results suggested that H2B ubiquitylation is enriched in both the introns and exons of *RP *genes, and that this particular pattern could be related to the high splicing activity of this group of intron-containing genes.

To determine whether chromatin structure contributed to the distribution of H2BK123ub1 at intron-containing genes, we utilized published data sets [42] to measure nucleosome occupancy in introns and exons of the these genes (Figure 2 D, E, F). Consistent with observations in *C. elegans *and human [10,43], we found that nucleosome occupancy in all intron-containing genes was reduced in the intron compared to flanking exons (Figure 2D). In addition, the overall nucleosome occupancy in both the introns and exons of *RP *genes (Figure 2E) was generally lower than in the introns of non-*RP *genes (Figure 2F). This is consistent with the observation that highly transcribed genes have lower histone occupancy [44]. We validated this nucleosomal pattern at several intron-containing *RP *genes using a highly accurate, probe-based quantitative PCR protocol. The results showed that both H2B and H3 levels were reduced in the introns of these genes compared to exons, indicating that overall nucleosome occupancy is lower in introns (Figure 2 G, H).

**Figure 3:**
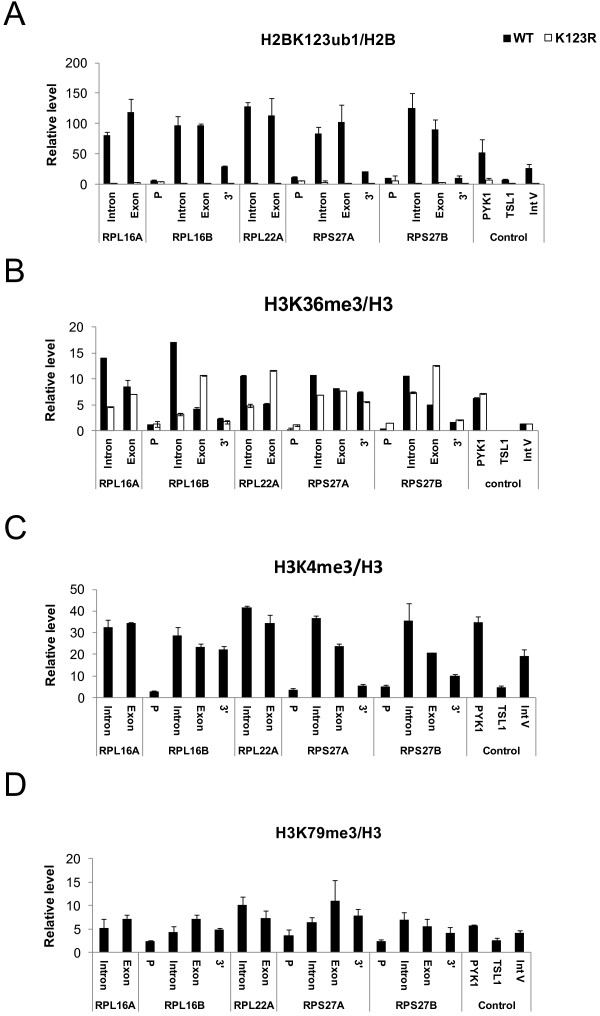
**H2BK123ub1, H3K36me3, H3K4me3, and H3K79me3 levels in introns and exons of ribosomal protein genes**. (A) ChDIP was performed to detect the level of HA-ubiquitin tagged Flag-H2B. Antibodies against (B) H3K36me3 (C) H3K4me3 and (D) H3K79me3 were used in ChIP to detect the levels of these three marks. Probe-based PCR primers were designed to amplify promoter (P), intron, exon, and 3' intergenic regions for the genes analyzed. H2BK123ub1 levels were normalized to H2B, and H3K36me3, H3K4me3 and H3K79me3 levels were normalized to H3. In (B) the levels of H3K36me3 were also measured in an *htb-K123R *mutant (K123R) that lacks H2BK123ub1. Error bars represent the standard deviation of at least three independent experiments.

Several reports have provided evidence for the existence of a special chromatin organization that marks introns and exons in the *C. elegans *and human genomes [5-7,9,10]. One of the key findings was that nucleosomes were enriched in exons and decorated with histone modifications that are associated with active transcription, such as H3K36me3. In contrast, nucleosome occupancy and the same histone modifications were generally depleted in introns. These data suggested that there is a functional connection between chromatin organization and the regulation of pre-mRNA splicing. Our results showing differential nucleosome occupancy between introns and exons in yeast are consistent with the analyses of intragenic nucleosome occupancy in worms and humans [5,10]. However, lower nucleosome occupancy in introns might not fully account for the differences in chromatin structure between introns and exons. Another report found that the 5' introns of human genes were enriched for 10 different histone modifications, including H2B ubiquitylation, and that this enrichment was separable from nucleosome occupancy [12]. Because the ChDIP assay used to detect H2BK123ub1 involves the internal normalization of H2BK123ub1 to H2B, our data support the view that this histone modification is also enriched in yeast introns compared to exons despite the lower overall occupancy of nucleosomes in introns (Figure 2G, H; Figure 3A). Furthermore, our results suggest that the elevated level of H2BK123ub1 in the introns of *RP *genes might provide a signal for co-transcriptional splicing.

Next, we addressed whether the sharp peak of H2BK123ub1 observed at the 3' intron-exon boundary of *RP *genes (Figure 2B) was a special feature of the chromatin structure at these regions. We noted that the average nucleosome occupancy in *RP *gene introns appeared to be lowest just before the 3' splice site, gradually increasing towards the 3' intron-exon boundary and extending into the 5' region of exon 2 (Figure 2E). By comparing the profiles of H2BK123ub1 and nucleosome occupancy at *RP *genes (Figure 2B, E), we conclude that the peak of H2BK123ub1 at the 3' intron-exon boundary is probably not the consequence of increased nucleosome occupancy in this region. Thus, our analysis suggests that the 3' intron-exon boundary of *RP *genes could contain a signal that facilitates H2BK123ub1 deposition or prevents its turnover.

### Loss of H2B ubiquitylation alters the distribution of H3K36 trimethylation in introns

The regulation of H3K4, H3K36, and H3K79 methylation by H2BK123ub1 raised the question of whether these marks would also be present in introns as well as exons. The published genome-wide datasets of histone modifications in yeast do not have the resolution to map the distributions of H3K4, H3K36, and H3K79 methylation at intron-exon boundaries [38]. Thus, to address this question, we used conventional ChIP to measure the levels of H3K36me3, H3K4me3, and H3K79me3 at the intron and 3' exon of the same *RP *genes used to analyze H2BK123ub1 (Figure 3). When compared to the levels of H3 at these same locations, it is clear that these three H3 marks are also enriched in introns (Figure 3B, C, D), similar to the enrichment of H2BK123ub1 (Figure 3A). The analysis of H3K36me3 levels in human and *C. elegans *suggested that H3K36me3 was enriched in exons and depleted in introns [7]; however, this difference vanished after H3K36me3 levels were normalized to nucleosome occupancy [10]. This emphasizes that it is essential to consider histone occupancy when comparing histone modification profiles across various genomic regions. Interestingly, in the absence of H2BK123ub1, H3K36me3 levels were significantly reduced in several *RP *gene introns (*RPL16A, RPL16B *and *RPL22A*) and increased in exons (*RPL16B, RPL22A*, and RPS27B) (Figure 3B). Because both H3K36me3 and H2BK123ub1 mark gene-coding regions, it is tempting to speculate that a dynamic balance between the distributions of the two histone marks contributes to the expression of these intron-containing genes. This notion is consistent with previous findings that altered levels of H2B ubiquitylation affect the levels of H3K36 methylation at the *GAL1 *gene [16,18]. Although H3K4me3 and H3K79me3 were also enriched in both introns and exons, we found that H3K4me3 was present at higher levels in introns compared to H3K79me3 (Figure 3C and 3D), perhaps reflecting the 5' bias in the distribution of H3K4me3 coupled with the typical 5' location of *RP *introns. Together, the data suggest that H2BK123ub1 and its downstream marks of H3K36me3, H3K4me3, and H3K79me3 are part of a chromatin architecture that defines *RP *gene introns.

### H2B ubiquitylation genetically interacts with RNA processing factors

**Figure 4:**
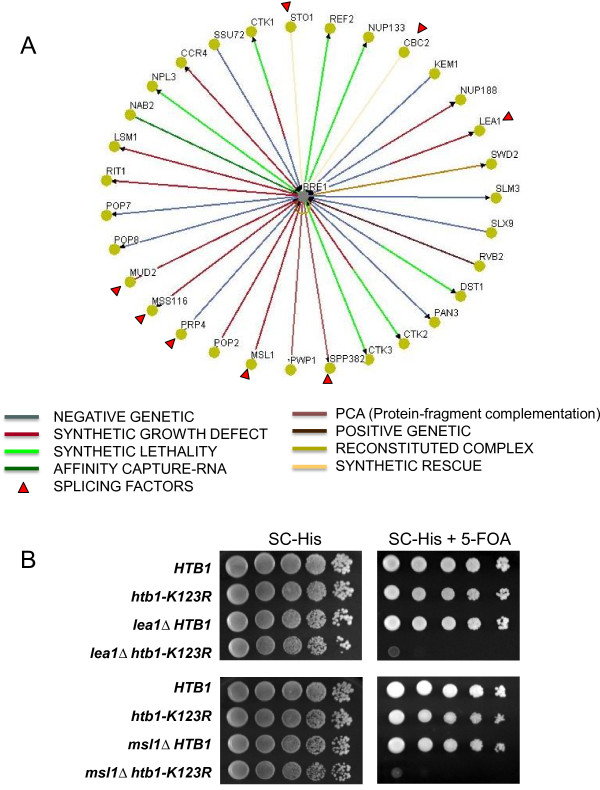
**Genetic interactions between the Bre1/H2B ubiquitylation and pre-RNA splicing pathways**. (A) Synthetic genetic interactions of *bre1Δ *were derived from BioGRID, and its interactions with RNA processing mutants were selected and displayed using the Osprey network visualization system [47]. Colored lines connect *bre1Δ *to mutations in genes leading to synthetic interactions (either positive or negative). The red triangle indicates genes that function in pre-mRNA splicing. (B) Growth analysis of double mutant cells of *lea1Δ htb-K123R *and *msl1Δ htb-K123R*. Cells carrying a *URA3 *plasmid expressing wild type *HTB1 *were transformed with a *HIS3 *plasmid carrying *HTB1 *or *htb-K123R*. After selection, transformants were grown at 30°C in SC-histidine medium for 24 hours. Cells were then spotted in 10-fold serial dilution onto SC-histidine plates or SC-histidine plates containing 1 mg of 5-FOA/ml, and the plates were incubated at 30°C for 2-3 days. Cells that were unable to lose the wild type *HTB1 *gene failed to grow on 5-FOA.

To determine if the distribution of H2BK123ub1 across intron-containing *RP *genes played a functional role in the regulation of mRNA splicing, we first asked if *BRE1*, which encodes the E3 ligase that mediates H2BK123ub1 [45,46], showed genetic interactions with genes for RNA processing factors. To test this, we examined genetic interaction data for a *bre1Δ *deletion mutant from BioGRID [47], which listed 370 interactions. After sorting the interaction data with Osprey, a Network Visualization System [47], we found that *bre1Δ *showed interactions with mutations in genes encoding 32 RNA processing factors, 8 of which are involved in pre-mRNA splicing (Figure 4A). Intriguingly, the data showed that *bre1Δ *conferred a synthetic growth defect (negative genetic interaction) when combined with mutations in the genes encoding the U1 snRNP component, Mud2; the U2 snRNP components, Spp382, Lea1 and Msl1; and the U4/U6-U5 snRNP complex member, Prp4. On the other hand, deletion of the capping complex genes, *STO1 *and *CBC2*, rescued the growth defect of *bre1Δ*. Because *BRE1 *genetically interacts with genes encoding many factors related to the regulation of RNA processing, this finding suggests that its activity in H2B ubiquitylation could play a role in pre-mRNA splicing.

To further address whether H2B ubiquitylation was responsible for the observed *bre1Δ *interactions with mutations in genes encoding RNA processing factors, we combined an *htb-K123R *mutation, which abolished H2B ubiquitylation, with the following mutations: a deletion of *MUD2*, which encodes a component of the pre-mRNA-U1 snRNP [48-50]; a deletion of *SAC3 *or *EDC2*, two genes encoding factors with roles in mRNA export and mRNA decapping [51,52]; and a deletion of *LSM1*, which encodes a protein involved in degradation of cytoplasmic mRNAs [48-50]. We found that the absence of H2BK123ub1 had no effect on the growth of *mud2, edc2Δ, sac3Δ *and *lsm1Δ *mutants (Additional file 1, Figure S5). However, when *htb-K123R *was combined with deletions of *LEA1 *and *MSL1*, whose products facilitate U2 snRNA association with pre-mRNA [29,30], we found a synthetic lethal phenotype (Figure 4B). A likely interpretation of these genetic interactions is that Bre1-mediated H2B ubiquitylation is functionally linked to U2 snRNP assembly.

## Discussion

The positioning of histone modifications at genes has been associated with numerous co-transcriptional processes ranging from initiation to elongation to 3' end processing. In this report, we investigated the genome-wide distribution of H2B monoubiquitylation in budding yeast. We found that H2BK123ub1, a mark of active transcription, is predominantly localized across gene coding regions, consistent with its postulated roles in transcription elongation [20,22,53]. The mark was also proportional to transcription rate, a likely consequence of the association of the H2B ubiquitylation machinery with elongating RNA polymerase II [32,54,55]. Both of these features are similar to the distribution of H2B monoubiquitylation in the human genome, supporting the view that both the regulation of this histone modification and its functional roles in transcription are evolutionarily conserved [34,56]. We also found a correlation between the levels of H2BK123ub1 and gene length, with the mark enriched on long genes. Because long genes frequently contain cryptic transcription initiation sites in their coding regions, this correlation supports the view that the presence of H2BK123ub1 contributes to the restoration of chromatin structure during transcription elongation to suppress the utilization of these sites [20].

A novel finding of our studies was the discovery that H2BK123ub1 is present in both the introns and exons of the yeast ribosomal protein genes which accounts for the majority of splicing events in yeast cells. Bioinformatic analyses of genome-wide data sets from worms and humans revealed that exons are enriched with active histone modifications while introns are generally depleted of these same marks [5,7,9]. This bias has been attributed primarily to chromatin architecture, with nucleosome occupancy higher in exons compared to introns [5-7,9,10]. Using published data sets on nucleosome occupancy in yeast [42], we found that yeast introns also have reduced nucleosome levels in introns compared to exons. However, this difference does not correlate with a reduction in H2BK123ub1 levels in the introns of the *RP *genes, which account for ~90% of the splicing activity of intron-containing genes [41]. The finding that H2BK123ub1 is a feature of yeast introns is similar to a recent finding, again from bioinformatic analysis of chromatin modification data sets, that H2B ubiquitylation is one of 10 marks of 5' introns in humans [12]. Most yeast genes with introns contain a single intron that is located close to the 5' end of the coding region [57-59]. Thus, the presence of H2B ubiquitylation in the 5' introns of human genes may reflect a similarity in the chromatin architecture of promoter-proximal introns between yeast and human intron-containing genes. We also found that H2BK123ub1 peaked at the 3' intron-exon boundary, particularly in the *RP *genes. We speculate that the chromatin structure of 3' intron-exon boundaries in these genes could carry a signal that enhances the accessibility of the enzymatic machinery that mediates H2B ubiquitylation (Rad6-Bre1) [27,28]. Alternatively, enzymes that target H2B for de-ubiquitylation (Ubp8 and Ubp10) [15,29] could be preferentially prevented from associating with these regions. It is currently unclear what feature of chromatin architecture at these boundaries regulates either the deposition or removal of H2B ubiquitylation. Likewise, it is not known if this distinct chromatin structure plays a role in transcriptional regulation, including pre-mRNA splicing.

H2B ubiquitylation controls the methylation of three lysine residues in histone H3 *in trans*. A comparison of the genome-wide distributions of H2BK123ub1, H3K4me3, H3K79me2/me3, and H3K36me3 showed that the four modifications occupy distinct regions in genes. As previously reported, H3K123ub1 and H3K79me3 co-localize across coding regions, while H2BK123ub1 and H3K79me2 show an anti-correlation at intergenic regions [8]. H3K4me3 is localized predominantly at 5' gene regions, while H3K36me3, like H2BK123ub1, spreads across coding regions, but with a more pronounced enrichment at the 3' end of genes. How H2BK123ub1 controls these particular distribution patterns remains an area of intense investigation [16,18,24,60-63]. H3K4me3 and H3K79me3, like H2BK123ub1, were also present in the introns of five ribosomal protein genes that were analyzed, and all of these marks were separable from nucleosome occupancy. Together, the results are similar to the reported presence of H2B ubiquitylation and H3K79me3 in the 5' introns of humans [12].

Unlike the situation in humans and worms [6,7,12], H3K36me3 is present in both the introns and exons of yeast genes. The restricted presence of H3K36me3 in exons in higher eukaryotic genomes has been correlated with the regulation of alternative mRNA splicing [6,7,28]. These observations suggest two possible roles for H3K36me3 in pre-mRNA splicing, and specifically in the regulation of pre-RNA splicing. First, H3K36me3 could mark exons as a part of a gene structure and along with *cis*-splicing elements facilitate the decision of whether to include a specific exon [6,12]. Alternatively, H3K36me3 could act as an anchor site for recruiting splicing factors that regulate alternative mRNA splicing [7,28]. However, in the yeast genome, the majority of intron-containing genes contain a single intron, and the regulation of splicing efficiency is thus more important than alternative mRNA splicing. It has been suggested that splicing efficiency is a function of the rate of RNA polymerase II elongation. This scenario is supported by a recent finding that RNAP II pauses transiently around the 3'end of introns and that this pause coincides with splicing factor recruitment [64]. Thus, the presence of histone marks in both introns and exons might promote splicing efficiency by controlling RNA polymerase II elongation. The finding that H2BK123ub1 levels are enhanced at 3' intron-exon boundaries could provide a mechanism to couple RNAPII elongation to the recruitment of splicing factors. Moreover, the dynamic relationship between the levels of H2BK123ub1 and H3K36me3 in introns and exons supports a redundant mechanism to ensure optimal RNAP II elongation, in turn promoting efficient pre-mRNA splicing.

Because the loss of H2B ubiquitylation, H3K4/K79 methylation, or H3K36 methylation does not compromise cell viability, the histone modifications cannot play an essential role in splicing. We suggest that the presence of these marks in introns, together with reduced nucleosome occupancy in these regions, are part of a chromatin architecture that facilitates the recognition of exons and introns by splicing regulators. Further support for this mechanism comes from the observation that a synthetic lethal phenotype resulted from combining an *htb-K123R *mutation with deletions of genes with roles in pre-mRNA splicing, specifically in U2 splicesome assembly. For example, the histone modifications might serve as binding sites for proteins that in turn interact with splicing factors. Such a scenario has been proposed for H3K4me3 and the Chd1 protein, which contains a chromodomain that recognizes the methyl mark and interacts with the U2 snRNP complex in both humans and yeast to promote efficient splicing [65-67].

## Conclusion

In summary, the co-transcriptional formation of H2BK123ub1 leads to its spread across gene coding regions. This mark in turn defines the distribution of H3K4 tri-methylation at the 5' end of coding regions and H3K79 tri-methylation toward the center of coding regions during the processes of transcription initiation and elongation. H2BK123ub1 also marks introns, and its presence in these regions leads to the presence of H3K4me3 and H3K79me3 at the introns of several ribosomal protein genes examined. The presence of H2BK123ub1 also influences the distribution of H3K36me3 across coding regions, particularly in introns. As a consequence, we suggest that this coordinated pattern of histone marks along transcribed regions facilitates the recognition of exons and introns and allows for efficient co-transcriptional pre-mRNA processing.

## Methods

### Yeast strains and growth conditions

Strains used in this study are listed in Additional file 1, Table S3. Yeast cultures were grown to mid-log phase in YPD medium (2% yeast extract; 2% peptone; 2% glucose) at 30°C for all ChIP-chip and growth studies.

### Chromatin Immunoprecipitation

ChIP was performed as described previously (Kao *et al*., 2004) with minor modifications. Chromatin pellets from formaldehyde fixed cells were lysed by glass bead vortexing for 30 min at 4°C. For all ChIP experiments analyzing individual genes, cell lysates were digested with 160 U of Micrococcal nuclease (MNase) per 100 ml of cells for 15 min (Nuclease S7, Roche Applied Science, Taiwan). The following antibodies were used with the equivalence of 10 OD units of cell lysate: FLAG, 20 μl (M2-F3165; Sigma-Aldrich, St. Louis, MO); HA, 5 μl (3F10; Roche Applied Science, Taiwan); H3, 4 μl (ab1791; Abcam, Cambridge, England); H3K4me3, 2 μl (ab8580; Abcam); H3K79me3, 10 μl (ab 2621, Abcam); and H3K36me3, 4 μl (ab9050, Abcam). Antibodies were prebound to protein A or protein G sepharose or dynabeads^®^. Purified DNAs were analyzed by probe-based real-time quantitative PCR on a Roche LightCycler^® ^480 Real-Time PCR System. Probe numbers are listed in Additional file 1, Table S4 and probes were ordered from Roche Universal Probe Library, Roche Applied Science, Taiwan. The IP/Input ratios of H2BK123ub1, H3K4me3, H3K36me3, and H3K79me3 were normalized to the IP/Input ratio of *INT-V *sequences as described previously [32].

### ChDIP-chip hybridization to oligonucleotide arrays

Chromatin pellets from formaldehyde fixed cells were lysed by glass bead vortexing for 30 min at 4°C, and the cell lysates were sonicated on a Branson Sonifier. ChDIP to measure H2BK123ub1 was performed as described previously [20,31] using a 2 step sequential ChIP. Briefly, the first ChIP was performed using anti-FLAG antibody, and the immune complexes were eluted with FA-lysis buffer containing 200 μg/ml of 3 × FLAG peptide. One-tenth of the eluate from the first ChIP was reserved for "input", and anti-HA antibody (12C5A; Roche) was added to a final concentration of 15 μg/ml to the remaining eluate. The immune complexes were then eluted with 1% SDS/50 mM Tris (pH 8.0) and represent "IP". After reversal of the crosslinks and purification, the IP and input DNA were amplified according to the Affymetrix protocol. IP and input samples from two biological replicates were hybridized to an Affymetrix 1.0R *S. cerevisiae *microarray, which comprised over 3.2 million probes covering the entire genome at 5 bp resolution.

### ChDIP-chip data analysis

#### Preprocessing and normalization

Two biological replicates for each paired ChIP (HA ubiquitin modified Flag-H2B; denoted by H2Bub1) and control (the level of H2B genomewide) were included in the experiments. Signals for ChIP-chip were extracted by the TileMap algorithm [68], in which perfect match (PM) only intensities were used. For each probe, the log ratio of intensities of ChIP and control were normalized using the quantile normalization method [69].

#### Averaged fold enrichments of all genes, intergenic regions and other regions of interest (in log_2 _scale)

*IP_ir _*and *C_ir _*denoted the PM value of probe *i *of ChIP and control arrays, and *r *denoted the *r*th replicate, where *r *= 1, 2. For each probe *i*, the replicated and normalized values were outputted to compute their averaged log ratios by the formula:

$$1∕2\left[log_{2}\left(IP_{i1}∕C_{i1}\right)+log_{2}\left(IP_{i2}∕C_{i2}\right)\right]$$

These averaged log ratios for all probes (including 4802 genes with assigned transcription rates) were allocated, via a code, to a genomic locus (ORF) and a 5' or 3' intergenic region, which were mapped evenly to the 20^th^-60^th^, 1^th^-20^th^, 60^th ^-80^th ^bins on the x-axis, respectively. In each bin, we averaged all probes of each gene, then averaging over all genes to result in the averaged intensity, which was plotted on top of the 5' intergenic, 'Average Gene' and the 3' intergenic regions to depict their fold enrichments. The averaged fold enrichments of whole genome, coding regions (ORF), intergenic regions and silent chromatin regions: *HM *mating type cassettes, telomeres and rDNA, were also computed. Similarly, the fold enrichment of all genes grouped by transcription rate, by gene length, and certain subgroups of interest were calculated, then smoothed by a moving window with size 5 (the averaged intensity of every five adjacent bins) [70], and plotted.

#### Analysis of intron-containing genes

##### Averaged fold enrichment of 5'exon-intron-3'exon (in log_2 _scale)

For intron-containing genes with defined transcription rates, each of their 5' exon (denoted as Exon 1), intron (Intron), and 3' exon (Exon 2) sequences were allocated to the 1^st ^-10^th ^, 10^th ^-40^th ^and 40 ^th ^-80^th ^bins, and averaged fold enrichments were calculated over genes. For the few multiple-intron-containing genes, the multiple copies of Exon 1-Intron-Exon 2 sequences were aligned by each Intron and allocated accordingly. Averaged fold enrichments (allocated to the middle point of each bin such as 2.5) were then calculated over genes and smoothed by a moving window with size 5, except for the first two and the last two bins of Exon 1, Intron, and Exon 2.

##### Averaged nucleosome occupancy

Nucleosome occupancy was defined as the percentage of DNA bound to a nucleosome, following the analysis in [42]. Scaled occupancy levels of nucleosomes in Exon 1 (Intron or Exon 2) ranging from 0 to 100% were weight averaged over 211-intron containing genes, proportional to the length of each Exon 1 (Intron or Exon 2), to yield the averaged nucleosome occupancy.

### Accession number

Raw sequencing data are available at the NCBI Gene Expression Omnibus (GEO)

(Accession number: GSE34325)

## Authors' contributions

CFK, MAO and SLB conceived of the study. CFK performed the ChDIP experiments and SLB supported the reagents and facilities for the ChDIP-chip experiments. CFK and MAO wrote the manuscript. CFK, CHP, WCH and CYL performed the gene-specific ChIP experiments. YCL, ABF and CH made yeast strains. LT and THC designed the experiments for the study of *bre1Δ *genetic interactions. GSS design, supervise and wrote the Genome-wide analysis. JHW, YJS and KWC assembled the genome-wide data set and carried out the analyses. All authors read and approved the final manuscript.

## Supplementary Material

Additional file 1 **This file contains Supplementary Figure S1-S5 and Supplementary table S1-S5**. The supplementary figures include: Figure S1. Validation of H2B ubiquitylation levels at various genomic regions; Figure S2. Comparison of the genomic distributions of H2BK123ub1, H3K4me3, H3K36me3, and H3K79me3; Figure S3. Comparison of the genomic distributions of H2BK123ub1 and H3K79me2/me3; Figure S4. Comparison of the regional distributions of H2BK123ub1 and H3K79me2/me3; Figure S5. H2B ubiquitylation does not interact with genes that function in U1 (*MUD2*), RNA export (*SAC3*), RNA decapping (*EDC2*) and RNA degradation (*LSM1*). The supplementary tables include: Table S1 Classification of genes by transcription rate or gene length; Table S2 Classification of highly transcribed genes by gene length; Table S3 S. cerevisiae strains; Table S4 Primers for probe based quantitative PCR; Table S5 Primers for quantitative PCR. Click here for file
