# Supplementary material for: H2B ubiquitylation is part of chromatin architecture that marks exon-intron structure in budding yeast
Source: BMC Genomics. 2011 Dec 22;12:627. doi: 10.1186/1471-2164-12-627 (PMC3274495; doi:10.1186/1471-2164-12-627)
Supplement: Additional file 1 — This file contains Supplementary Figure S1-S5 and Supplementary table S1-S5. The supplementary figures include: Figure S1. Validation of H2B ubiquitylation levels at various genomic regions; Figure S2. Comparison of the genomic distributions of H2BK123ub1, H3K4me3, H3K36me3, and H3K79me3; Figure S3. Comparison of the genomic distributions of H2BK123ub1 and H3K79me2/me3; Figure S4. Comparison of the regional distributions of H2BK123ub1 and H3K79me2/me3; Figure S5. H2B ubiquitylation does not interact with genes that function in U1 (MUD2), RNA export (SAC3), RNA decapping (EDC2) and RNA degradation (LSM1). The supplementary tables include: Table S1 Classification of genes by transcription rate or gene length; Table S2 Classification of highly transcribed genes by gene length; Table S3 S. cerevisiae strains; Table S4 Primers for probe based quantitative PCR; Table S5 Primers for quantitative PCR. [file 1471-2164-12-627-S1.PDF]

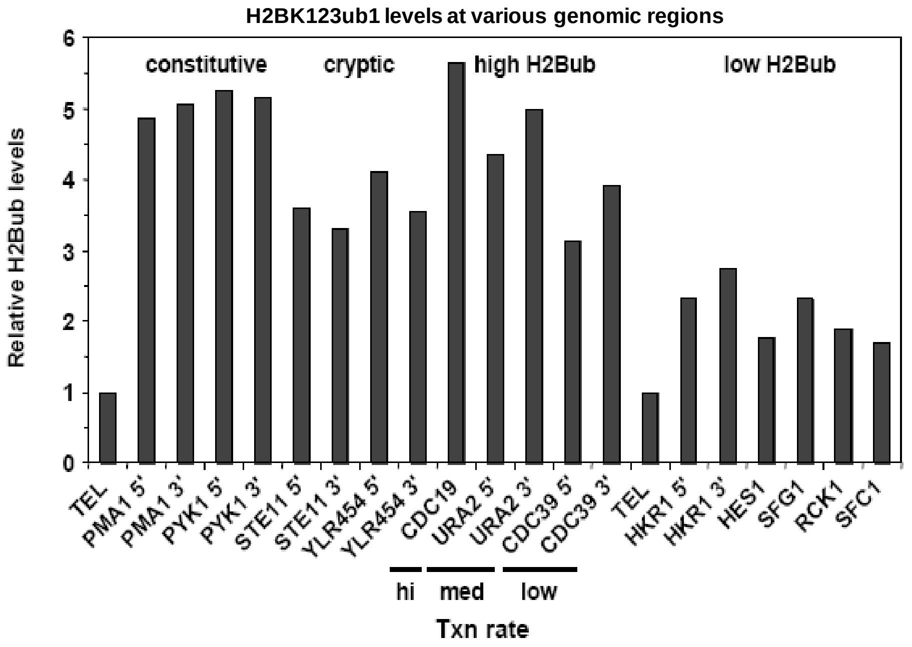


**Figure S1. Validation of H2B ubiquitylation levels at various genomic regions.** To validate the genome-wide data, H2BK123ub1 ChDIP was sequentially performed with anti-Flag and anti-HA antibodies in strain CFK1548 after growth at 30°C in YPD medium. Quantitative real time PCR was used to measure the level of H2BK123ub1 at the indicated genomic regions. Constitutive: genes with constitutive expression; cryptic: genes with internal transcription initiation sites in their coding regions; high H2Bub: genes with high levels of H2BK123ub1 grouped according to high, medium, and low transcription rates; low H2Bub: genes or regions with low levels of H2BK123ub1.

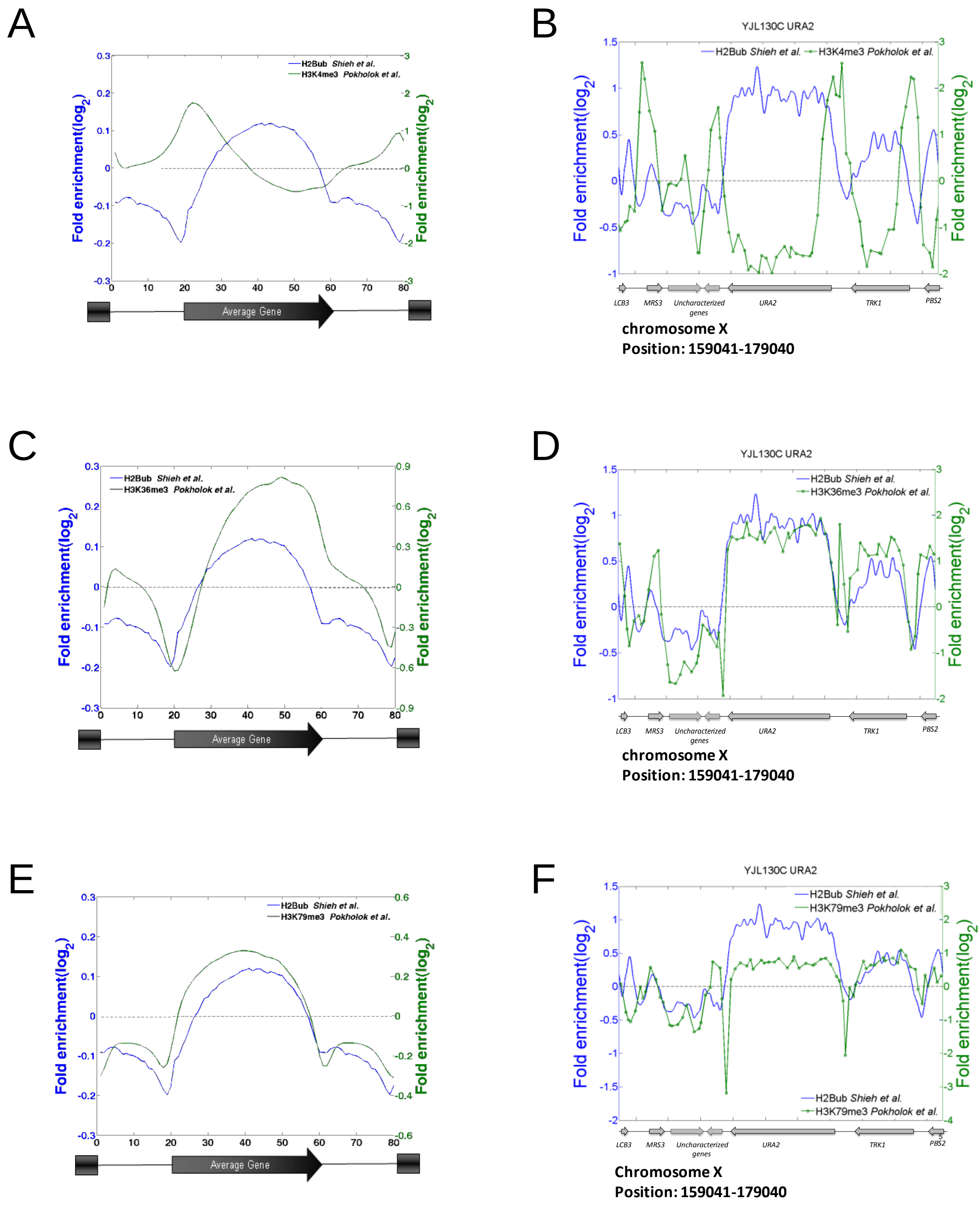


**Figure S2. Comparison of the genomic distributions of H2BK123ub1, H3K4me3, H3K36me3, and H3K79me3.** Distribution of H2BK123ub1 and (A) H3K4me3; (C) H3K36me3; and (E) H3K79me3 across a composite plot of the average gene . Distribution of H2BK123ub1 and (B) H3K4me3, (D) H3K36me3, and (F) H3K79me3 along a locus on chromosome X: 159041-179040. The H3K4me3, H3K36me3, and H3K79me3 data came from Pokholok *et al.* [1].

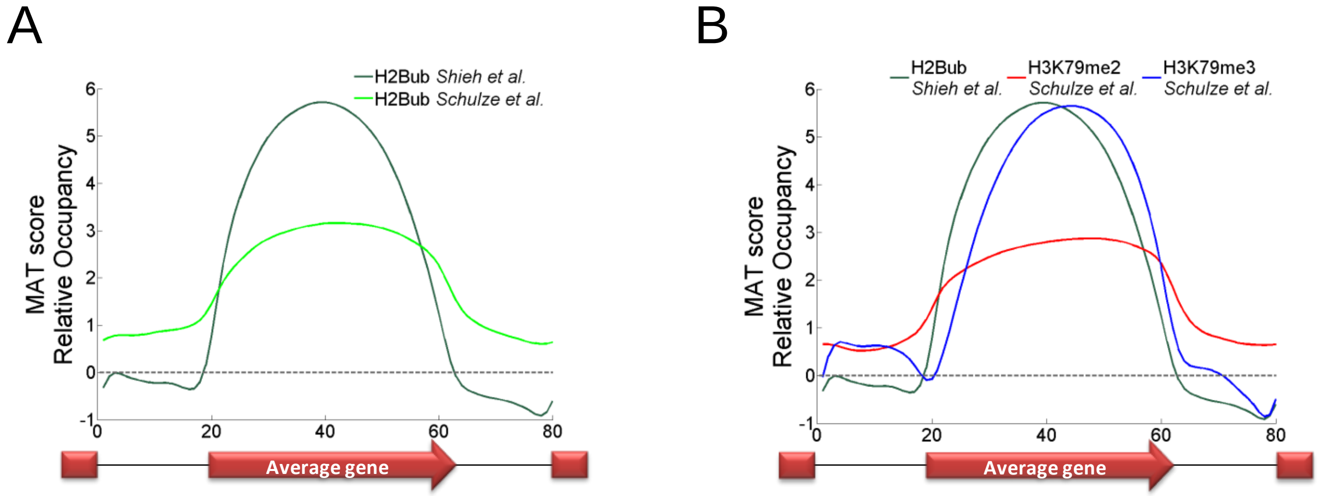


**Figure S3. Comparison of the genomic distributions of H2BK123ub1 and H3K79me2/me3.** (A) Comparison of H2BubK123ub1 genome-wide profiles between the data in this ms. and the data from Schulze *et al.* [2] that resulted from using a polyclonal antibody against H2BK123ub1. The two data sets were analyzed using the MAT algorithm [3], and a composite of the average gene profile is shown. (B) Comparison of the H2BubK123ub1 genome-wide profile in this ms. to the genome-wide profiles of H3K79me2 and H3K79me3 from Schulze *et al*. [2]. The data sets were analyzed and displayed as in (A).

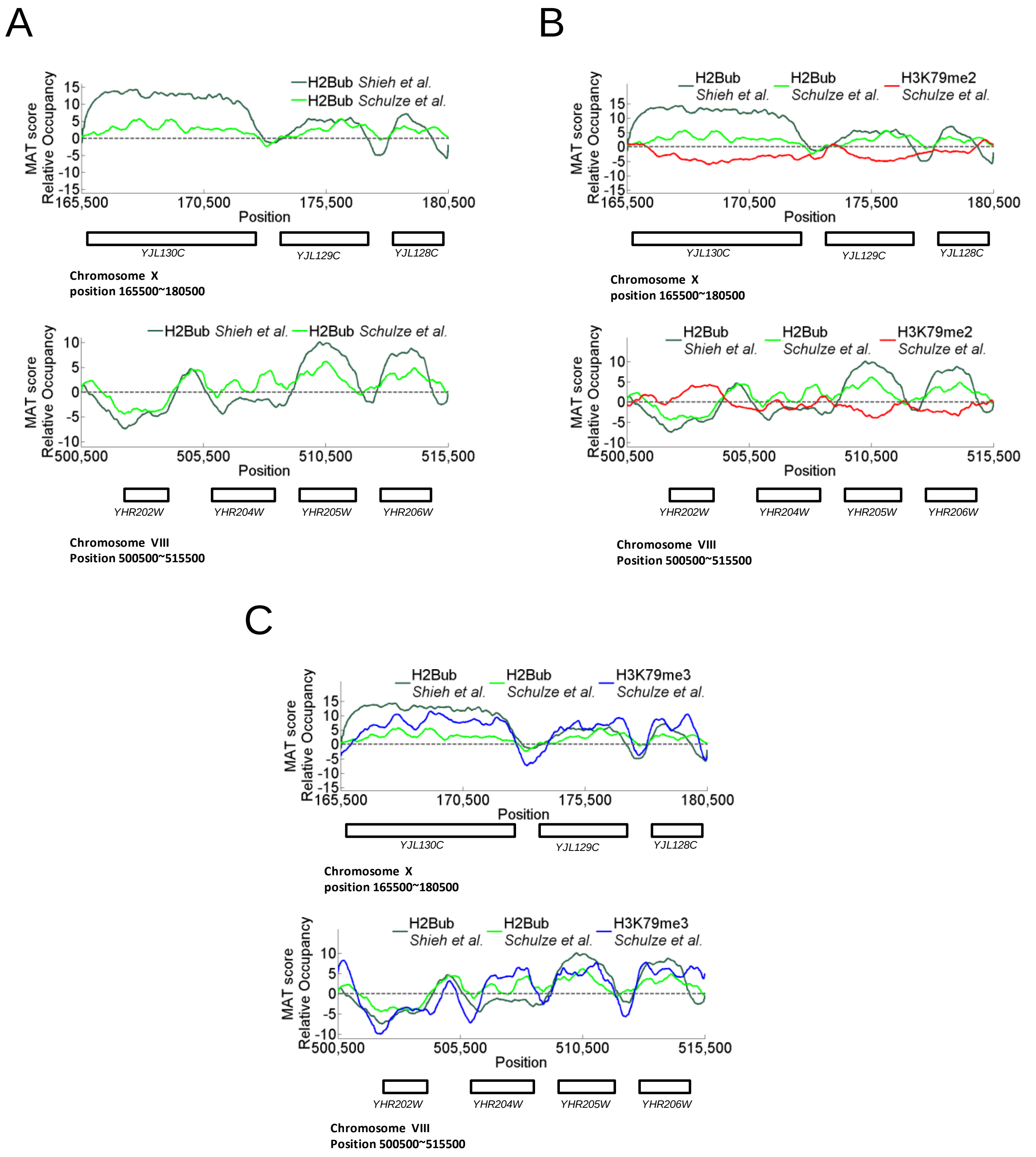


**Figure S4. Comparison of the regional distributions of H2BK123ub1 and H3K79me2/me3.**
(A) Overlay of the genome-wide profiles of H2BK123ub1 from this ms. and from Schulze *et al.* at two genomic regions. The MAT scores of the indicated data (dark green: *Sheih et al.*; light blue: *Schulze et al.*) were plotted along the X axis of the indicated genomic positions for chromosomes 8 and 10 against the MAT score representing the relative occupancies on the Y axis. Coding regions are indicated as open bars. (B) Overlay of the profiles of H2BK123ub1 and H3K79me2 at sample genomic regions plotted as in (A). (C) Overlay of the profiles of H2BK123ub1 and H3K79me3 at sample genomic regions plotted as in (B).

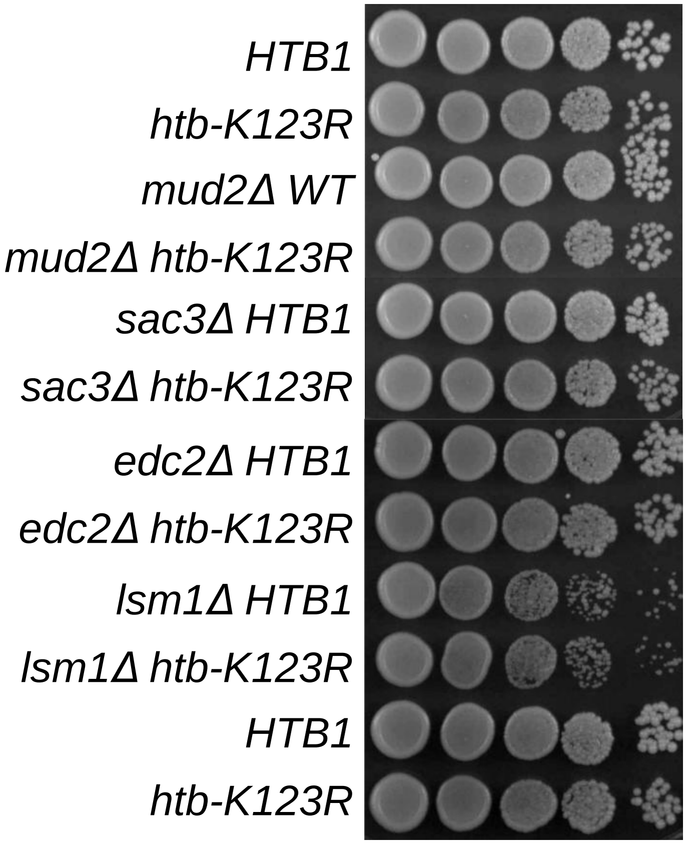


**Figure S5. H2B ubiquitylation does not interact with genes that function in U1 (*MUD2*), RNA export (*SAC3*), RNA decapping (*EDC2*) and RNA degradation (*LSM1*).** Growth analysis of double mutant cells of *mud2*Δ *htb-K123R, sac3*Δ *htb-K123R, edc2*Δ *htb-K123R,* and *lsm1*Δ *htb-K123R* . WT or mutant strains with *mud2*Δ*, sac3*Δ*, edc2*Δ or *lsm1*Δ carrying a *HIS3* plasmid expressing wild type *HTB1 or htb-K123R* were spotted in 10-fold serial dilution onto YPD plates and the plates were incubated at 30℃ for 3 days.

**References**

1. Pokholok DK, Harbison CT, Levine S, Cole M, Hannett NM, et al. (2005) Genome-wide Map of Nucleosome Acetylation and Methylation in Yeast. Cell 122: 517-527
2. Schulze JM, Jackson J, Nakanishi S, Gardner JM, Hentrich T, et al. (2009) Linking cell cycle to histone modifications: SBF and H2B monoubiquitination machinery and cell-cycle regulation of H3K79 dimethylation. Mol Cell 35: 626-641.
3. Johnson WE, Li W, Meyer CA, Gottardo R, Carroll JS, et al. (2006) Model-based analysis of tiling-arrays for ChIP-chip. Proc Natl Acad Sci U S A 103: 12457-12462.

**Table S1. Classification of genes by transcription rate or gene length**

| | <1 mRNA/hr | 1-4 mRNA/hr | 4-16 mRNA/hr | 16-50 mRNA/hr | > 50 mRNA/hr | Total | % |
|---|---|---|---|---|---|---|---|
| <500 | 161 | 180 | 125 | 38 | 38 | 542 | 11.29% |
| 500-1000 | 278 | 501 | 351 | 68 | 96 | 1294 | 26.95% |
| 1000-2000 | 500 | 855 | 410 | 93 | 31 | 1889 | 39.34% |
| 2000-3000 | 215 | 335 | 93 | 15 | 4 | 662 | 13.79% |
| 3000-4000 | 83 | 129 | 35 | 4 | 1 | 252 | 5.25% |
| 4000-5000 | 33 | 51 | 13 | 1 | 0 | 98 | 2.04% |
| 5000-6000 | 9 | 12 | 10 | 3 | 0 | 34 | 0.71% |
| >6000 | 14 | 11 | 4 | 2 | 0 | 31 | 0.65% |
| Total | 1293 | 2074 | 1041 | 224 | 170 | 4802 | 100% |
| % | 26.93% | 43.19% | 21.68% | 4.66% | 3.54% | 100% | |

Data source: Holstege et al., 1998

**Table S2. Classification of highly expressed genes genes by gene length**

| | gene number | % of all lengths |
|---|---|---|
| gene >16 mRNA and shoter than 2000bp | 364 | 92.39% |
| gene >16 mRNA and shoter than 1000bp | 240 | 60.91% |

Data source: Holstege et al., 1998

**Table S3 *S. cerevisiae* strains**

| Strains | Genotype | Source |
|---|---|---|
| YZS276 | *MAT***a** *hta1-htb1⊿::LEU2, hta2-htb2⊿, leu2-3,-112 his3-11,-15 trp1-1 ura3-1 ade2-1 can1-100 (pZS145 HTA1-Flag-HTB1 CEN HIS3)* | (Xiao et al., 2005) |
| YZS277 | *MAT***a** *hta1-htb1⊿::LEU2, hta2-htb2⊿, leu2-3,-112 his3-11,-15 trp1-1 ura3-1 ade2-1 can1-100 (pZS146 HTA1-Flag-htb1-K123R CEN HIS3)* | (Xiao et al., 2005) |
| CFK1548 | *MATa, hta1-htb1⊿::LEU2, hta2-htb2⊿, ura3-1, trp1-1, leu2-3,-112, his3-11, ade2-1, can1-100, GAPDH-HA-UBI4::URA3 <pZS145 HTA1-Flag-HTB1, CEN, HIS3>* | (Fleming et al., 2008) |
| CFK1549 | *MATa, hta1-htb1⊿::LEU2, hta2-htb2⊿, ura3-1, trp1-1, leu2-3,-112, his3-11, ade2-1, can1-100, GAPDH-HA-UBI4::URA3 <pZS146 HTA1-Flag-htb1-K123R, CEN, HIS3>* | (Fleming et al., 2008) |
| YPC001 | *MAT***a** *hta1-htb1⊿::LEU2, hta2-htb2⊿, leu2-3,-112 his3-11,-15 trp1-1 ura3-1 ade2-1 can1-100 mud2::KanMX (pZS145 HTA1-Flag-HTB1 CEN HIS3)* | This study |
| YPC002 | *MAT***a** *hta1-htb1::LEU2⊿, hta2-htb2⊿, leu2-3,-112 his3-11,-15 trp1-1 ura3-1 ade2-1 can1-100 mud2Δ::KanMX (pZS146 HTA1-Flag-htb1-K123R CEN HIS3)* | This study |
| YPC003 | *MAT***a** *hta1-htb1⊿::LEU2, hta2-htb2⊿, leu2-3,-112 his3-11,-15 trp1-1 ura3-1 ade2-1 can1-100 sac3Δ::KanMX (pZS145 HTA1-Flag-HTB1 CEN HIS3)* | This study |
| YPC004 | *MAT***a** *hta1-htb1⊿,::LEU2, hta2-htb2⊿, leu2-3,-112 his3-11,-15 trp1-1 ura3-1 ade2-1 can1-100 sac3Δ::KanMX (pZS146 HTA1-Flag-htb1-K123R CEN HIS3)* | This study |
| YPC005 | *MAT***a** *hta1-htb1⊿::LEU2, hta2-htb2⊿, leu2-3,-112 his3-11,-15 trp1-1 ura3-1 ade2-1 can1-100 edc2Δ::KanMX (pZS145 HTA1-Flag-HTB1 CEN HIS3)* | This study |
| YPC006 | *MAT***a** *hta1-htb1⊿,::LEU2, hta2-htb2⊿, leu2-3,-112 his3-11,-15 trp1-1 ura3-1 ade2-1 can1-100 edc2Δ::KanMX (pZS146 HTA1-Flag-htb1-K123R CEN HIS3)* | This study |
| YPC007 | *MAT***a** *hta1-htb1⊿,::LEU2, hta2-htb2⊿, leu2-3,-112 his3-11,-15 trp1-1 ura3-1 ade2-1 can1-100 lsm1Δ::KanMX (pZS146 HTA1-Flag-htb1-K123R CEN HIS3)* | This study |
| YPC008 | *MAT***a** *hta1-htb1⊿,::LEU2, hta2-htb2⊿, leu2-3,-112 his3-11,-15 trp1-1 ura3-1 ade2-1 can1-100 lsm1Δ::KanMX (pZS146 HTA1-Flag-htb1-K123R CEN HIS3)* | This study |
| YPC009 | *MAT***a** *hta1-htb1⊿,::LEU2, hta2-htb2⊿, leu2-3,-112 his3-11,-15 trp1-1 ura3-1 ade2-1 can1-100, lea1Δ::KanMX (pZS146 HTA1-Flag-htb1-K123R CEN HIS3)* | This study |

| | | |
|---|---|---|
| YPC0010 | *MAT***a** *hta1-htb1⊿,::LEU2, hta2-htb2⊿, leu2-3,-112 his3-11,-15 trp1-1 ura3-1 ade2-1 can1-100 msl1Δ::KanMX (pZS146 HTA1-Flag-htb1-K123R CEN HIS3)* | This study |
| YAF120 | *MATa, hhf2-hht2::NAT, hta1-htb1::HPH, hht1-hhf1::KAN, hta2-htb2::NAT, ura3-52, trp1Δ2, leu2-3,-112, his3-11, ade2-1, can1-100, GAL1-YLR454w::TRP1 <pRS315-HTA1-Flag-HTB1, HHT1-HHF1>* | (Fleming et al., 2008) |
| YAF121 | *MATa, hhf2-hht2::NAT, hta1-htb1::HPH, hht1-hhf1::KAN, hta2-htb2::NAT, ura3-52, trp1Δ2, leu2-3,-112, his3-11, ade2-1, can1-100, GAL1-YLR454w::TRP1 <pRS315-HTA1-Flag-htb1-K123R, HHT1-HHF1>* | (Fleming et al., 2008) |
| YAF122 | *MATa, hhf2-hht2::NAT, hta1-htb1::HPH, hht1-hhf1::KAN, hta2-htb2::NAT, ura3-52, trp1Δ2, leu2-3,-112, his3-11, ade2-1, can1-100, GAL1-YLR454w::TRP1 <pRS315-HTA1-Flag-HTB1, hht1-K4A-HHF1>* | (Fleming et al., 2008) |
| YAF124 | *MATa, hhf2-hht2::NAT, hta1-htb1::HPH, hht1-hhf1::KAN, hta2-htb2::NAT, ura3-52, trp1Δ2, leu2-3,-112, his3-11, ade2-1, can1-100, GAL1-YLR454w::TRP1 <pRS315-HTA1-Flag-HTB1, hht1-K79A -HHF1>* | (Fleming et al., 2008) |
| YAF125 | *MATa, hhf2-hht2::NAT, hta1-htb1::HPH, hht1-hhf1::KAN, hta2-htb2::NAT, ura3-52, trp1Δ2, leu2-3,-112, his3-11, ade2-1, can1-100, GAL1-YLR454w::TRP1 <pRS315-HTA1-Flag-HTB1, hht1-K79A -HHF1>* | (Fleming et al., 2008) |

References

Fleming, A.B., Kao, C.F., Hillyer, C., Pikaart, M., and Osley, M.A. (2008). H2B ubiquitylation plays a role in nucleosome dynamics during transcription elongation. Mol Cell *31*, 57-66.

Xiao, T., Kao, C.F., Krogan, N.J., Sun, Z.W., Greenblatt, J.F., Osley, M.A., and Strahl, B.D. (2005). Histone H2B ubiquitylation is associated with elongating RNA polymerase II. Mol Cell Biol *25*, 637-651.

**Table S4 Primers for probe-based quantitative PCR**

| | Roche Universal Probe Library Probe number | | |
|---|---|---|---|
| PYK1 | (# 163) | F | aattgtacccaggtagaccattg |
| | | R | tggtaccagttctgatttctgg |
| TSL1 | (# 7) | F | ttaaaccccggaaaaatggt |
| | | R | tccgtgggaatattggaaga |
| Int V | (# 72) | F | gctgtaaaatttgaccgatttgt |
| | | R | actacggccccatattctga |
| RPL16A-intron | (# 28) | F | gcactaataattgaatgtgttttcct |
| | | R | acgctgtgcactatgagcaa |
| RPL16B-intron | (# 39) | F | cgttgttattgatggtacgtaaaaa |
| | | R | caacacatttcgcaggattatt |
| RPL22B-intron | (# 153) | F | tgttaatagcagtaggccagacat |
| | | R | tgagatgaaatcatccttgtcg |
| RPS27A-intron | (# 31) | F | agataagtcgcaaaagaaaagaataca |
| | | R | cttgcctctcttggtgcaa |
| RPS27B-intron | (# 94) | F | ttggagcacaaatgctcact |
| | | R | gggttgggtaggatacatcaga |
| RPL16A-exon | (# 145) | F | ttccgttggttggaaatacg |
| | | R | gcggatgaaacctttctcttt |
| RPL16B-exon | (# 142) | F | aggtcagatccgccgaata |
| | | R | aagcagaggagaccttttttgg |
| RPL22A-exon | (# 86) | F | tgaagatggtaccgttgttactg |
| | | R | ttggttcttcttcaagtacttctttg |
| RPL22B-exon | (# 55) | F | agttatcaaaaccttaaccgttgac |
| | | R | gaatatgaagccggatcgaa |
| RPS27A-exon | (# 131) | F | tgcgaatcctgctcaaca |
| | | R | tgaaagatgtaccctcagaaagc |
| RPS27B-exon | (# 129) | F | atttgttgcacccaactgct |
| | | R | ttgggccttgaaccaaagt |
| RPL16B P | (# 29) | F | gccaacgggtgaaaaaca |
| | | R | ttcaggttctaggagcgttctc |
| RPL16B 3’ | (# 165) | F | acggtcaagtaattcaaaattgg |
| | | R | ttcgatacaacttgggtgagag |

| | | | |
|---|---|---|---|
| RPS27A P | (# 47) | F | cggcttaaaattttggtattcatt |
| | | R | cacatcggaggaacacagaa |
| RPS27A 3’ | (# 14) | F | tctccttttctagcttgtggaac |
| | | R | agcggccaaaacacagatta |
| RPS27B P | (# 49) | F | aaggaaggtgtgttcgcatt |
| | | R | tagatgggtggcaaacaaaa |
| RPS27B 3’ | (# 55) | F | agtaggtcggcaggttcattat |
| | | R | cctacggattgtgcctttg |

**Table S5 Primers for quantitative PCR**

| RP Gene primers | | |
|---|---|---|
| RPL16A | F | TTC CGT TGG TTG GAA ATA CG |
| | R | GCG GAT GAA ACC TTT CTC TTT |
| RPL16B | F | AGG TCA GAT CCG CCG AAT A |
| | R | AAG CAG AGG AGA CCT TTT TGG |
| RPL22A | F | TGA AGA TGG TAC CGT TGT TAC TG |
| | R | TTG GTT CTT CTT CAA GTA CTT CTT TG |
| RPL22B | F | AGT TAT CAA AAC CTT AAC CGT TGA C |
| | R | GAA TAT GAA GCC GGA TCG AA |
| RPS27A | F | TGC GAA TCC TGC TCA ACA |
| | R | TGA AAG ATG TAC CCT CAG AAA GC |
| RPS27B | F | ATT TGT TGC ACC CAA CTG CT |
| | R | TTG GGC CTT GAA CCA AAG T |
